# Histone Crosstalk Directed by H2B Ubiquitination Is Required for Chromatin Boundary Integrity

**DOI:** 10.1371/journal.pgen.1002175

**Published:** 2011-07-21

**Authors:** Meiji Kit-Wan Ma, Carol Heath, Alan Hair, Adam G. West

**Affiliations:** Institute of Cancer Sciences, College of Medical, Veterinary, and Life Sciences, University of Glasgow, Glasgow, United Kingdom; Massachusetts General Hospital, Howard Hughes Medical Institute, United States of America

## Abstract

Genomic maps of chromatin modifications have provided evidence for the partitioning of genomes into domains of distinct chromatin states, which assist coordinated gene regulation. The maintenance of chromatin domain integrity can require the setting of boundaries. The HS4 insulator element marks the 3′ boundary of a heterochromatin region located upstream of the chicken *β-globin* gene cluster. Here we show that HS4 recruits the E3 ligase RNF20/BRE1A to mediate H2B mono-ubiquitination (H2Bub1) at this insulator. Knockdown experiments show that RNF20 is required for H2Bub1 and processive H3K4 methylation. Depletion of RNF20 results in a collapse of the active histone modification signature at the HS4 chromatin boundary, where H2Bub1, H3K4 methylation, and hyperacetylation of H3, H4, and H2A.Z are rapidly lost. A remarkably similar set of events occurs at the HSA/HSB regulatory elements of the *FOLR1* gene, which mark the 5′ boundary of the same heterochromatin region. We find that persistent H2Bub1 at the HSA/HSB and HS4 elements is required for chromatin boundary integrity. The loss of boundary function leads to the sequential spreading of H3K9me2, H3K9me3, and H4K20me3 over the entire 50 kb *FOLR1* and *β-globin* region and silencing of *FOLR1* expression. These findings show that the HSA/HSB and HS4 boundary elements direct a cascade of active histone modifications that defend the *FOLR1* and *β-globin* gene loci from the pervasive encroachment of an adjacent heterochromatin domain. We propose that many gene loci employ H2Bub1-dependent boundaries to prevent heterochromatin spreading.

## Author Summary

The transcription of genes in eukaryotes occurs within the context of chromatin, a complex of DNA, histone proteins, and regulatory factors. Whole-genome profiling of chromatin proteins and histones that are post-translationally modified has revealed that genomes are organized into domains of distinct chromatin states that coordinate gene regulation. The integrity of chromatin domains can require the setting of their boundaries. DNA sequences known as chromatin insulator or boundary elements can establish boundaries between transcriptionally permissive and repressive chromatin domains. We have studied two chromatin boundary elements that flank a condensed chromatin region located between the chicken *FOLR1* and *β-globin* genes, respectively. These elements recruit enzymes that mediate the ubiquitination of histone H2B. Histone H2B ubiquitination directs a cascade of so-called “active” histone modification events that favor chromatin accessibility. We observe a striking collapse of the active histone modification signature at both chromatin boundaries following the depletion of ubiquitinated H2B. This loss of boundary function leads to the comprehensive spreading of repressive chromatin over the entire *FOLR1* and *β-globin* gene region, resulting in gene silencing. We propose that chromatin boundaries at many gene loci employ H2B ubiquitination to restrict the encroachment of repressive chromatin.

## Introduction

There is growing consensus that the non-random chromosomal arrangement of genes in higher eukaryotes enables the sharing of specific chromatin environments that facilitate co-regulation.

Recent genomic profiling of histone modifications, chromatin factors and nuclear proximity in *Drosophila* and mammalian cells have revealed prevalent organization of genes into domains, or neighborhoods, of common chromatin state [1]–[5]. Genes taken out of their natural chromosomal environment become deregulated in a variety of human genetic diseases [6]. This so-called chromosomal position effect also underlies the variable expression of transgenes depending on their site of integration [7].

The maintenance of chromatin domain integrity can require the setting of boundaries. Boundaries not only allow the partitioning of gene regulation, but also may also maintain the concentration of factors required for heterochromatin structures and normal genome homeostasis [8]. Fixed chromatin boundaries can be established by DNA sequence elements called insulators, which function to protect genes from inappropriate signals emanating from their surrounding environment [9]–[12]. HS4 is a well characterized element that has served as a paradigm for the study of insulators in vertebrates. HS4 lies at a boundary between the chicken *β-globin* gene cluster and upstream region of condensed chromatin that is enriched in the epigenetic hallmarks of heterochromatin [13]–[15]. A 275 bp core of the HS4 element has two separable activities that functionally define insulators: it can block the action of an enhancer element on a linked promoter when positioned between the two and it can act as a barrier to chromosomal position effect silencing [16]–[18]. The enhancer blocking and barrier activities of HS4 involve different proteins and mechanisms and are separable in assay systems. The CTCF binding site footprint II (FII) is necessary and sufficient for enhancer blocking, but can be deleted from HS4 without affecting barrier activity [18]–[20]. HS4 requires a USF1/USF2 binding site (FIV) and three VEZF1 binding sites (FI, FIII and FV) for its barrier activity, which control histone modifications and DNA methylation, respectively [21]–[24].

HS4 manipulates histone modification signatures to counteract gene silencing [22], [24]. HS4 has been found to be persistently enriched in high levels of H3 and H4 acetylation, H3-lysine 4 methylation, H4-arginine 3 methylation and acetylated histone variant H2A.Z regardless of neighboring gene expression [13]–[14], [23], [25]. We proposed that the active histone modifications at HS4 collectively act as a chain terminator to heterochromatin assembly by interfering with the propagation of repressive histone modifications [24].

Given that chromosomal silencing has been shown to be processive and stable, we reasoned that the HS4 element needs to act as a constitutive barrier if it is to effectively shield the locus. In this study, we address a hypothesis that HS4 might recruit histone modifications that act as master controllers of the active chromatin state to facilitate barrier stability. Intense study in recent years has begun to unravel the complex language of crosstalk between histone modifications during the establishment of different chromatin states [26]. Principal among the active histone modifications is the monoubiquitination of H2BK120 (H2BK123 in *S. cerevisiae*), which is required for the tri-methylation of H3K4 [27]–[30]. H3K4me3 is a pivotal mark of the active chromatin state, by acting as a platform for the binding of multiple histone acetyltransferase, histone demethylase and nucleosome remodelling complexes [31]–[33]. We therefore investigate whether i) H2B ubiquitination directs a cascade of active histone modifications at the HS4 insulator, ii) this modification is required for its barrier activity, and iii) the integrity of the 3′ chromatin boundary of the condensed chromatin located upstream of the *β-globin* locus. We also extend our analysis to look at the 5′ chromatin boundary of the same condensed chromatin and its role in shielding the *FOLR1* gene locus.

## Results

### Histone ubiquitination is a mark of chromatin boundaries

**Figure 1:**
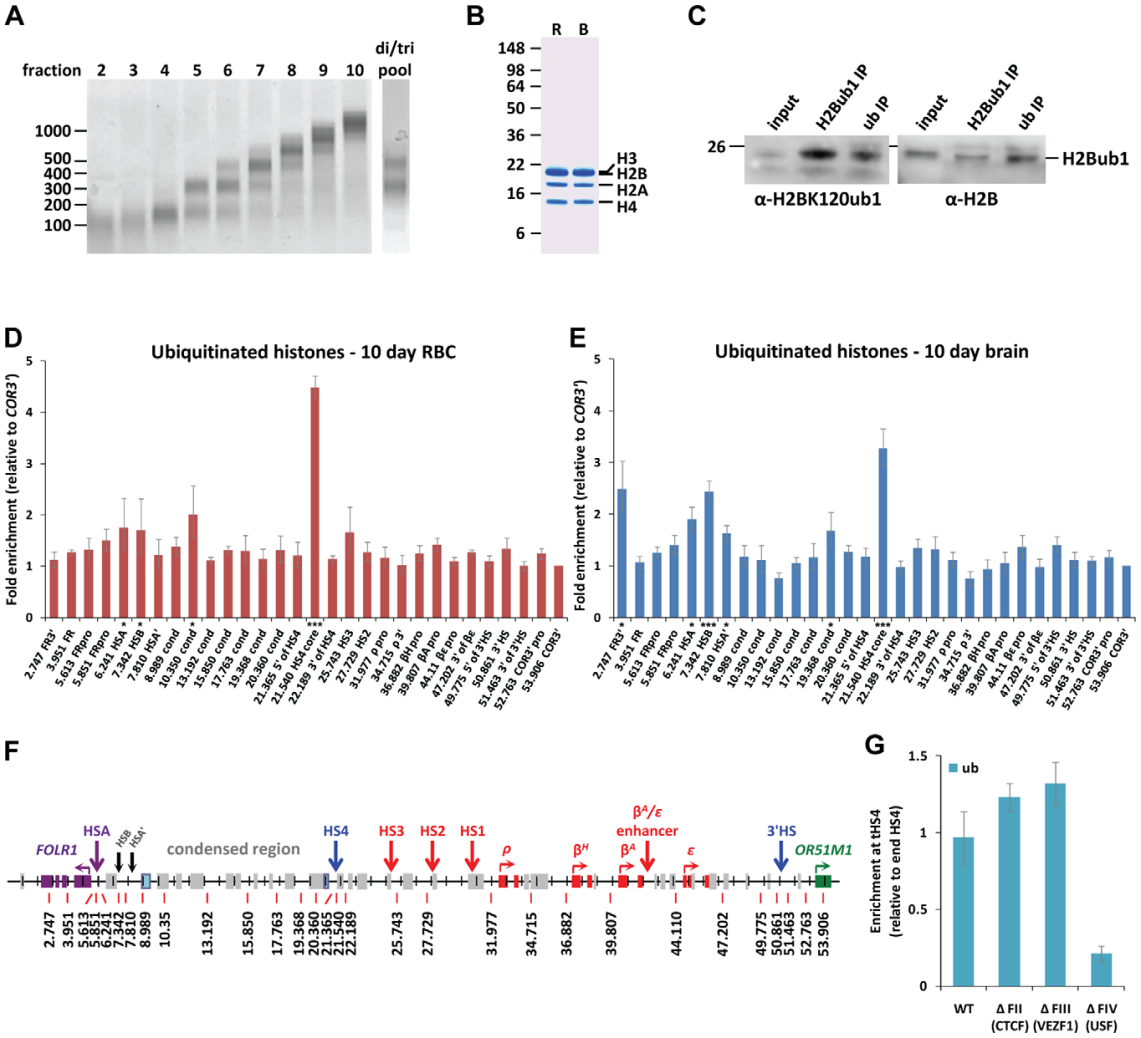
The HS4 insulator is enriched with ubiquitinated histones. A) Sucrose gradient fractionation of native MNase-digested nucleosomes. Fractions containing di- and tri- nucleosomes (e.g., 5–7) are pooled for ChIP analysis. B) SDS-PAGE analysis of histone purity in di/tri-nucleosome preparations from 10 day chick embryo red cells (R) and whole brain (B). C) Western blot analysis of mono-ubiquitinated H2B present in immunoprecipitates from 6C2 cell di/tri-nucleosomes. D, E) Native ChIP of histone ubiquitination at sites across the chicken *β-globin* gene neighborhood in 10 day chick embryo red cells (D) and whole brain (E). The enrichment of each sequence is normalized to the background observed at the downstream inactive *OR51M1* (*COR3′*) gene. Significant ChIP enrichments are represented by asterisks (⋆ =  p<0.01, ⋆⋆ =  p<0.001 and ⋆⋆⋆ =  p<0.0001). F) Scale map of the chicken *β-globin* gene neighborhood. The exons of the *FOLR1, β-globin* (*ρ, β^H^, β^A^ and ε*) and *OR51M1* genes are depicted by purple, red and green boxes, respectively. Grey boxes represent CR1 repeat sequences. Light blue boxes indicate ERV1 sequences. Vertical arrows show early/late erythroid (purple/red) or constitutive (blue) DNaseI hypersensitive regulatory elements. G) The USF site is required for histone ubiquitination at transgenic HS4 insulators stably integrated into 6C2 cells. Native ChIP enrichments normalized to the endogenous HS4 element.

We sought to address whether histone H2B ubiquitination plays a key role in establishing and maintaining the boundaries of a condensed heterochromatin-like domain that separates the *FOLR1* and *β-globin* gene loci. Firstly, we mapped the presence of ubiquitinated nucleosomes across 50 kb encompassing the chicken *β-globin* locus (Figure 1). We established native chromatin immunoprecipitation (N-ChIP) assays using nucleosomes prepared by micrococcal nuclease (MNase) digestion of chromatin in low salt conditions to ensure the retention of potentially unstable variant nucleosomes found at this locus [34]. We prepared di- and tri-nucleosomes using a range of MNase concentrations which ensured that they were representative of open and condensed chromosomal regions (Figure 1A, data not shown). The N-ChIP method strips away non-nucleosomal proteins (Figure 1B), which allows the analysis of ubiquitinated histones using anti-ubiquitin antibodies. The enrichment of nucleosomes containing the 25 kDa monoubiquitinated form of H2B was confirmed by western blotting (Figure 1C).

N-ChIP analysis of histone ubiquitination was performed on primary red blood cells (RBC) from 10 day chick embryos, in which the *β-globin* locus is highly transcriptionally active, but the 5′ folate receptor (*FOLR1)* locus is silent [15], [35]. We observe a striking enrichment of ubiquitinated histones specifically at the core HS4 insulator element (Figure 1D; p-value from student's t-test of enrichment  =  2e^−6^). Perhaps surprisingly, no enrichment of histone ubiquitination was observed at the promoters or enhancers of the highly active *β-globin* genes in RBCs. We also mapped histone ubiquitination in 10 day embryo whole brain tissue (Figure 1E), where both the *FOLR1* and *β-globin* genes are reported to be silent [15]. We also observe a specific enrichment of ubiquitinated histones specifically at the core HS4 insulator element in brain tissues (Figure 1E, p-value  =  6e^−5^). We also observe significant levels of histone ubiquitination at the *FOLR1* gene regulatory elements HSA and HSB in both 10 day embryo RBCs (Figure 1D, p-value  =  0.007) and whole brain (Figure 1E, p-value  =  2e^−5^). These elements are situated between the *FOLR1* gene and the condensed region and may harbor chromatin boundary activity.

We sought to determine which of the well characterized activities of the 275 bp core HS4 element are required for the recruitment of histone ubiquitination. We performed N-ChIP analyses of histone ubiquitination at HS4 insulators present on single copy transgenes stably integrated into the early erythroid CFU-E stage cell line 6C2 [18]. We find that transgenic HS4 insulators are enriched in histone ubiquitination at a level equivalent to the endogenous HS4 element (WT, Figure 1G). Histone ubiquitination is therefore likely to be recruited by one of the factors that mediate the insulator functions of the core HS4 element. We performed N-ChIP analysis of single copy transgenic HS4 elements that are mutated at the CTCF (FII), VEZF1 (FIII) or USF1 (FIV) binding sites. These mutations have been extensively characterized and disrupt HS4's ability to mediate enhancer blocking, protection from DNA methylation or active histone modification, respectively [19], [21], [24]. We find that the USF1/USF2 binding site, footprint IV, is required for the recruitment of histone ubiquitination (Figure 1G). This correlates with our previous finding that the USF site was also required for H3K4 methylation of the HS4 insulator [24].

### RNF20 is required for H2B ubiquitination and *trans* histone crosstalk

**Figure 2:**
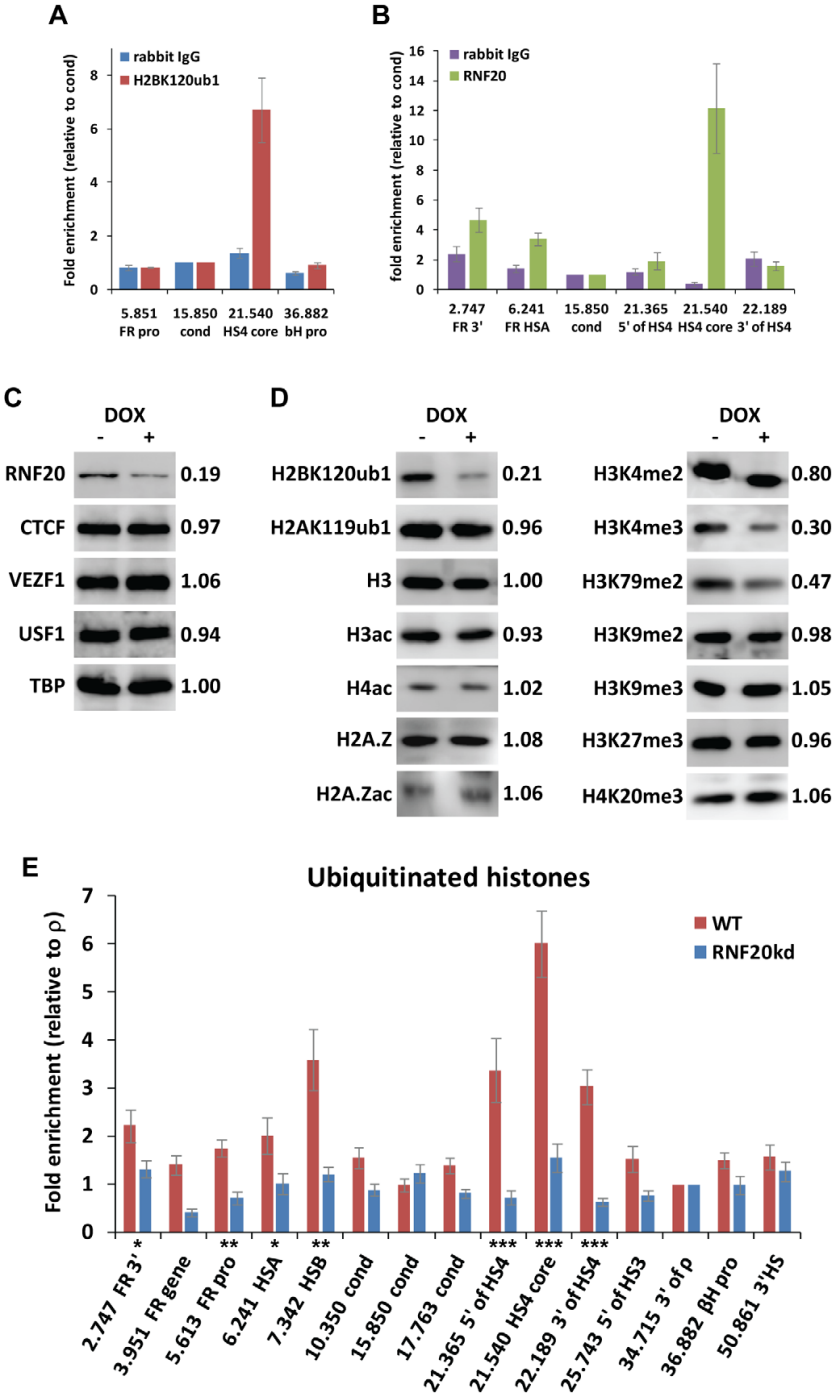
The ubiquitin ligase RNF20 mediates H2B ubiquitination at the HS4 insulator. Crosslinking ChIP analysis of (A) H2BK120ub1 and (B) RNF20 enrichment at the chicken *β-globin* locus in 6C2 cells. The enrichment of each sequence is normalized to the background observed at the condensed region (15.850). C–E) Analyses of early erythroid 6C2 cells following four days of doxycycline-induced knockdown of RNF20 expression. (C) Western blotting of whole cell extracts with (+) and without (−) doxycycline-induced RNF20 knockdown. The expression levels of each factor (relative to the loading control TBP) after RNF20 knockdown are shown. (D) Western blotting of histone modifications present on total nucleosomes with and without RNF20 knockdown. The levels of each modification after RNF20 knockdown (relative to unmodified H3 loading control) are shown. (E) Native ChIP of histone ubiquitination at sites across the chicken *β-globin* gene neighborhood in wild type (red bars) and RNF20 knockdown (blue bars) 6C2 cells. The enrichment of each sequence is normalized to the background observed downstream of the inactive *ρ-globin* gene. Significant changes in ChIP enrichments following RNF20 depletion are represented by asterisks (⋆ =  p<0.01, ⋆⋆ =  p<0.001 and ⋆⋆⋆ =  p<0.0001).

The histone ubiquitination that is enriched at the HS4 element may occur on any of the core histones. We anticipated that histone H2B is subject to this modification as HS4 is constantly enriched in methylated H3K4 [13], [24], and H2BK120 mono-ubiquitination is required for proper H3K4 methylation [27]–[30]. Using crosslinking ChIP with recently developed antibodies, we confirmed that the core HS4 insulator was enriched in H2BK120ub1 in the early erythroid CFU-E stage cell line 6C2 (Figure 2A). We were unable to detect any enrichment of H2AK119ub1 at HS4 or other *β-globin* sequences (not shown).

We sought to identify the E3 ligase responsible for H2Bub1 at the HS4 element so that the effects of depleting H2Bub1 could be studied. We used crosslinking ChIP analysis to show that RNF20 interacts with the core HS4 insulator element in erythroid cells (Figure 2B). Chicken RNF20 (BRE1A), is 90% identical to human RNF20/BRE1A, which is an E3 ligase responsible for efficient H2B ubiquitination (Figure S1A) [36]–[37]. The presence of RNF20 therefore suggests that this enzyme is required for the enrichment of H2BK120ub1 at the HS4 insulator.

Next, we investigated whether H2Bub1 levels can be depleted following RNAi of RNF20. It was important that we were able to knockdown RNF20 levels for prolonged periods as this would allow the study of progressive repression of the *β-globin* locus and insulated transgenes. This was achieved in 6C2 cells using a lentiviral vector system for doxycycline-regulated expression of miRNA-shRNA (Materials and methods, Figure S1). After four days of shRNA expression, RNF20 protein levels were reduced to ∼20% of wild type (Figure 2C). We saw little change in the whole cell protein levels of the HS4-binding proteins CTCF, VEZF1 or USF1. We studied the effect of this short term knockdown of RNF20 expression on a panel of histone modifications in total chromatin. We found that H2BK120ub1 levels in chromatin were reduced by ∼80% (Figure 2D). RNF20 knockdown did not affect H2AK119ub1 levels. This confirmed that chicken RNF20 is a H2B-specific ubiquitin E3 ligase like its Bre1 orthologs. The reduction of H2B ubiquitination resulted in substantial reductions in H3K4me3 (70%) and H3K79me2 (53%) levels, and a minor reduction in H3K4me2 (20%) (Figure 2D). This demonstrates that chickens also employ the same trans-histone crosstalk pathways observed in yeast and mammals [26]. H3K9acK14ac was slightly reduced (7%), but the levels of other modifications associated with active or repressive chromatin remained largely unchanged (Figure 2D).

To determine whether RNF20 was responsible for all the histone ubiquitination observed at the HS4 and *FOLR1* elements, we performed N-ChIP analysis across the *FOLR1* and *β-globin* region before and after RNF20 knockdown in 6C2 cells. Consistent with our observations in primary 10 day embryo tissues, we find that the HS4 insulator and the *FOLR1* HSA/HSB elements are substantially enriched in histone ubiquitination in 6C2 cells (Figure 2E). In addition, there is elevated histone ubiquitination across the *FOLR1* gene, which is highly active in 6C2 cells, consistent with co-transcriptional deposition (Figure 2E). We observed a substantial depletion of histone ubiquitination at the HS4 insulator and *FOLR1* HSA/HSB elements following four days of RNF20 knockdown (Figure 2E; p-values from student's t-test of difference between WT and RNF20kd are 2e^−5^ and 0.002, respectively). We observe similar profiles of RNF20-dependent H2Bub1 in 6C2 cells (Figure S2). The histone ubiquitination observed at HS4 and the *FOLR1* regulatory elements is therefore RNF20-dependent H2B monoubiquitination.

### Loss of H2B ubiquitination results in a collapse of the active chromatin signatures at the *FOLR1* and *β-globin* chromatin boundaries

The HS4 insulator is marked by an assemblage of histone modifications and variants typically associated with transcriptionally permissive open chromatin; H3K9acK14ac, H4K5acK8acK12acK16ac, H3K4me2, H3K4me3, H4R3me2as, H2A.ZK4acK7acK11ac, and H2BK120ub1 [13]–[14], [23] and this study). This active modification signature is a constant feature of HS4 in a variety of cell types irrespective of local gene expression. A very similar chromatin signature is observed at the *FOLR1* HSA/HSB regulatory elements in 6C2 cells. We hypothesize that H2Bub1 may be the keystone for the deposition of the active histone signature at these elements. We therefore performed N-ChIP analysis of active and repressive modifications across the 50 kb *β-globin* gene neighborhood following short term knockdown of H2B ubiquitination.

**Figure 3:**
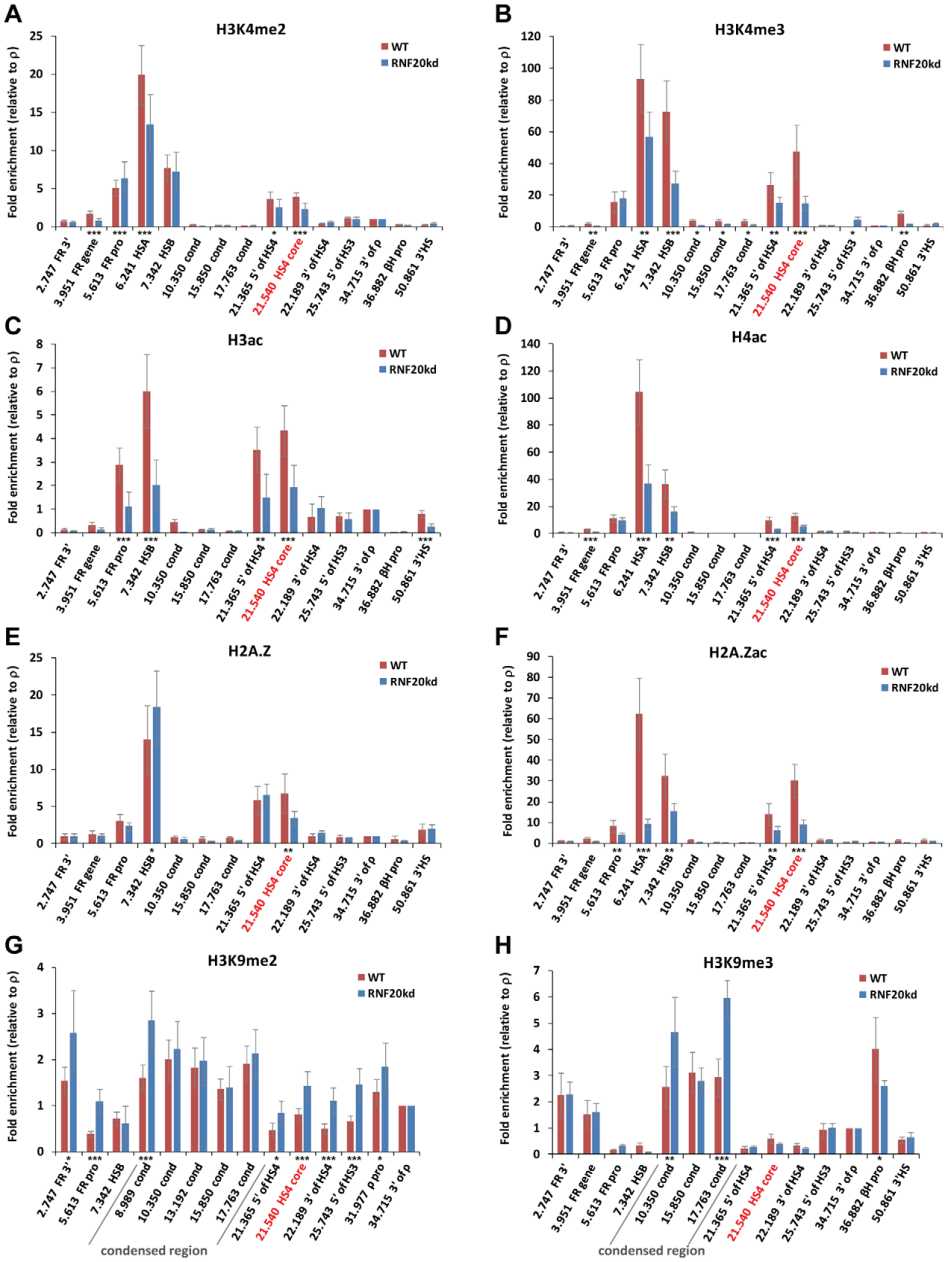
Loss of H2B ubiquitination results in a rapid loss of active histone modifications at the HS4 insulator. Native ChIP analyses of early erythroid 6C2 cells following four days of doxycycline-induced knockdown of RNF20 expression. Enrichments of A) H3K4me2, B) H3K4me3, C) H3K9acK14ac, D) H4K5acK8acK12acK16ac, E) H2A.Z, F) H2A.ZK4acK7acK11ac, G) H3K9me2 and H) H3K9me3 at sites across the chicken *β-globin* gene neighborhood in wild type (red bars) and RNF20 knockdown (blue bars) cells. The enrichment of each sequence is normalized to the background observed downstream of the inactive *ρ-globin* gene (34.715). Significant changes in ChIP enrichments following RNF20 depletion are represented by asterisks (⋆ =  p<0.05, ⋆⋆ =  p<0.01 and ⋆⋆⋆ =  p<0.005). The location of the core HS4 insulator (site 21.540) is highlighted in red. Sites within the condensed region are indicated in panels G and H.

We found that H3K4me2 and H3K4me3 enrichments at the HS4 insulator element were reduced by 40% and 70%, respectively, following RNF20 knockdown (21.540, Figure 3A and 3B). This is consistent with *trans* H2Bub1-H3K4me3 cross-talk occurring at HS4 nucleosomes. The depletion of H3K4me at HS4 is specific as the levels observed at the active *FOLR1* gene promoter remain unchanged (5.613, Figure 3A and 3B). Strikingly, the loss of H2Bub1 also considerably impacts the hyperacetylation of multiple histones at the HS4 insulator, with H3ac, H4ac and H2A.Zac reduced by 55%, 60% and 70%, respectively (21.540, Figure 3C, 3D and 3F). The depletion of histone acetylation at the HS4 insulator is in contrast to the relatively unchanged levels of histone acetylation in bulk chromatin (Figure 2D). We note that very similar depletions in active modifications are also observed at the *FOLR1* gene regulatory elements HSA/HSB. These regulatory elements may harbor functional properties similar to those of the HS4 insulator element. H2A.Z incorporation was mostly unaffected, but there was a 50% reduction in H2A.Z levels specifically at the core of the HS4 insulator (21.540, Figure 3E).

We investigated whether the depletion of H2Bub1 and the resulting loss of the active histone signatures at the HSA/HSB and HS4 elements affected the containment of the intervening condensed region. We determined that H3K9me2 and H3K9me3 are restricted to the condensed region upstream of HS4 in wild type 6C2 cells (8.9 to 17.7, Figure 3G and 3H, not shown). We find that after only four days of H2Bub1 depletion there is marked encroachment of H3K9me2 beyond the HS4 insulator. Significant H3K9me2 spreading into the *β-globin* locus is observed at all sites from the condensed region to the *ρ-globin* gene promoter (Figure 3G). Significant H3K9me2 spreading is also observed in the other direction, encompassing the *FOLR1* promoter and gene body. No encroachment of H3K9me3 is observed after short term depletion of H2Bub1, but there is considerable consolidation of this mark at the edges of the condensed region (Figure 3H). The heterochromatin associated mark H4K20me3 is also enriched in the condensed region and at the 3′ end of the *FOLR1* gene, but did not spread upon four days of RNF20 knockdown (Figure S3). Finally, we found that the gene silencing mark H3K27me3 was present at comparably low levels across the condensed region and *β-globin* locus in 6C2 cells, which did not alter upon RNF20 knockdown (data not shown). In summary, short term depletion of H2Bub1 is sufficient to disrupt H3K4me3 at the HSA/HSB and HS4 elements, which results in a rapid loss of multiple histone acetylation and chromatin boundary integrity. H3K9me2 appears as the first repressive mark to spread beyond the defective boundaries of the condensed heterochromatin region.

### Insulator protein complexes remain intact despite chromatin boundary failure

**Figure 4:**
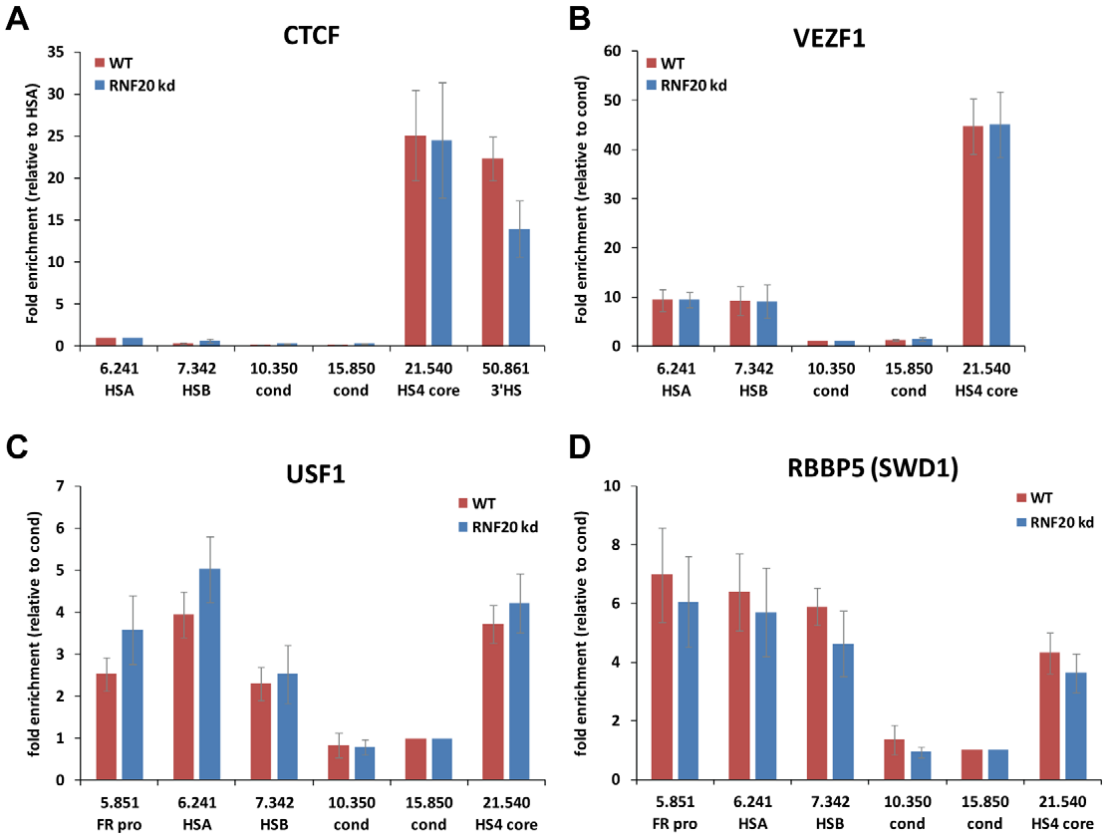
Loss of H2B ubiquitination does not disrupt the binding of insulator protein complexes at HS4. Crosslinking ChIP analysis of A) CTCF, B) VEZF1, C) USF1 and D) RBBP5 occupancy at the chicken *FOLR1*/*β-globin* loci in 6C2 cells before (red) and after (blue bars) the induction of RNF20 knockdown.

The comprehensive loss of active histone modifications at the HS4 boundary following RNF20 knockdown may be due to reduced binding of the insulator proteins that recruit histone modifying enzymes. We showed above that the expression of the insulator proteins is unaffected following RNF20 knockdown (Figure 2C). We therefore determined the binding of the insulator factors USF1, CTCF and VEZF1 to HS4 using crosslinking ChIP analysis before and after the loss of active modifications following RNF20 knockdown. We find that the binding of each factor is unaffected following RNF20 knockdown (Figure 4A–4C). We also discovered that the heterochromatin barrier factors VEZF1 and USF1 are also stably bound at the *FOLR1* HSA and HSB elements, which contain binding motifs for both factors (Figure 4B and 4C). The *FOLR1* region is not bound by the enhancer blocking and chromatin looping factor CTCF (Figure 4A). In summary, the disruption of insulator protein binding is not responsible for the comprehensive loss of active histone modifications at the HSA/HSB and HS4 elements.

We also addressed whether the depletion of H2Bub1 prevented the stable recruitment of histone methyltransferase (HMT) complexes that target H3-lysine 4. Existing models used to explain *trans*-tail crosstalk between H2Bub1 and H3K4me3 propose that the ubiquitination of H2B either regulates HMT residence by controlling nucleosome stability or creates a binding interface for HMT binding to chromatin (see discussion). We performed crosslinking ChIP analysis for RBBP5, a structural component of the SET1/COMPASS complex that interacts with USF1 [22]. We find that RBBP5 interacts with the HS4 insulator and the *FOLR1* regulatory elements, all of which are sites of H3K4me3. The binding of RBBP5 to the HS4 or HSA/HSB boundary elements is not significantly affected by RNF20 knockdown (Figure 4D). The loss of H3K4me3 upon H2Bub1 depletion is therefore not due to the decreased residence of the core SET1 complex at HS4.

### H2B ubiquitination is required for chromatin barrier activity

**Figure 5:**
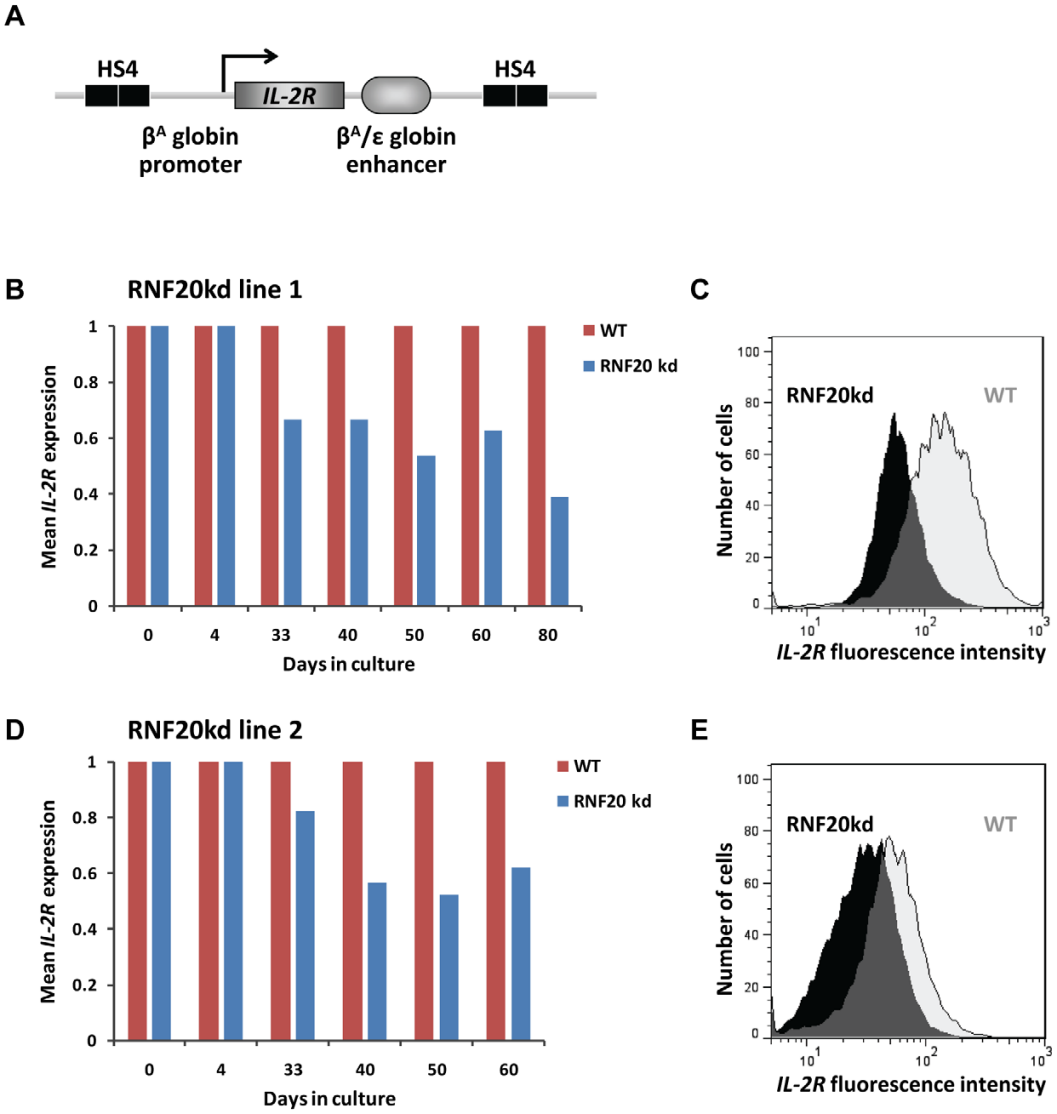
H2B ubiquitination is required for HS4 barrier activity. A) Schematic representation of the *IL-2R* reporter transgene flanked by double copy HS4 insulators. B–E) FACS analyses of IL-2R expression in two independent RNF20 knockdown lines over time in culture. B, D) Mean fluorescence intensity of IL-2R expression in two independently derived RNF20 knockdown lines (blue bars), relative to wild type parental cells (red bars). C, E) IL-2R fluorescence in the cell lines shown in panels (B) and (D) after 80 and 60 days of RNF20 knockdown, respectively, overlaid with that of parental wild type (WT) cells.

The ability of the HS4 element to shield genes from chromosomal position effect silencing in a wide variety of systems is well established [38]. This so-called barrier activity can be scored using a well established reporter transgene assay in erythroid cells [17]–[18], [24]. We used this assay to monitor the expression of a human IL-2R fragment from stably integrated transgenes (Figure 5A) using flow cytometry over time in culture. Non-insulated transgenes typically succumb to chromosomal silencing by 40–60 days of culture, whereas transgenes insulated by HS4 elements are able to maintain original levels of expression for 80 days and beyond [18]. We took extensively characterized stable lines that each contain a single copy of the *IL-2R* transgene flanked by paired 275 bp core HS4 insulators. It was previously determined that transgene expression from these cells remains constant beyond 80 days of culture, the transgenic HS4 insulators are bound by CTCF, USF1 and VEZF1 [21], and they are enriched in H3ac, H4ac, H3K4me [18], [24] and H2Bub1 (Figure 1G).

We transduced early passage *IL-2R* transgenic cells with lentiviruses that express RNF20 shRNA. The lentiviral miRNA-shRNA system we employed allowed the stable knockdown of RNF20 for at least sixty days (validated by Western blotting). We observed no change in 6C2 cell morphology and only a minimal reduction in cell doubling during this period (not shown). We found that four days of RNF20 knockdown had no effect on transgene expression (day 4, Figure 5B, 5D). The depletion of H2Bub1 therefore has little direct effect on the transcription rate of the transgene. However, transgene expression became progressively silenced with continued depletion of H2Bub1, with the IL-2R expression levels in independent transgenic lines falling by 50–60% after long term depletion (Figure 5B, 5D). This level of silencing is less than that observed when flanking insulators are absent or mutated [18], but is comparable to that observed in cells transfected with AUSF, a truncated form of USF1 that dominantly inhibits USF1 function [22]. Thus, constant H2B ubiquitination is required for HS4 to act as a stable barrier to chromosomal silencing.

### Loss of boundary function leads to comprehensive spreading of repressive chromatin

**Figure 6:**
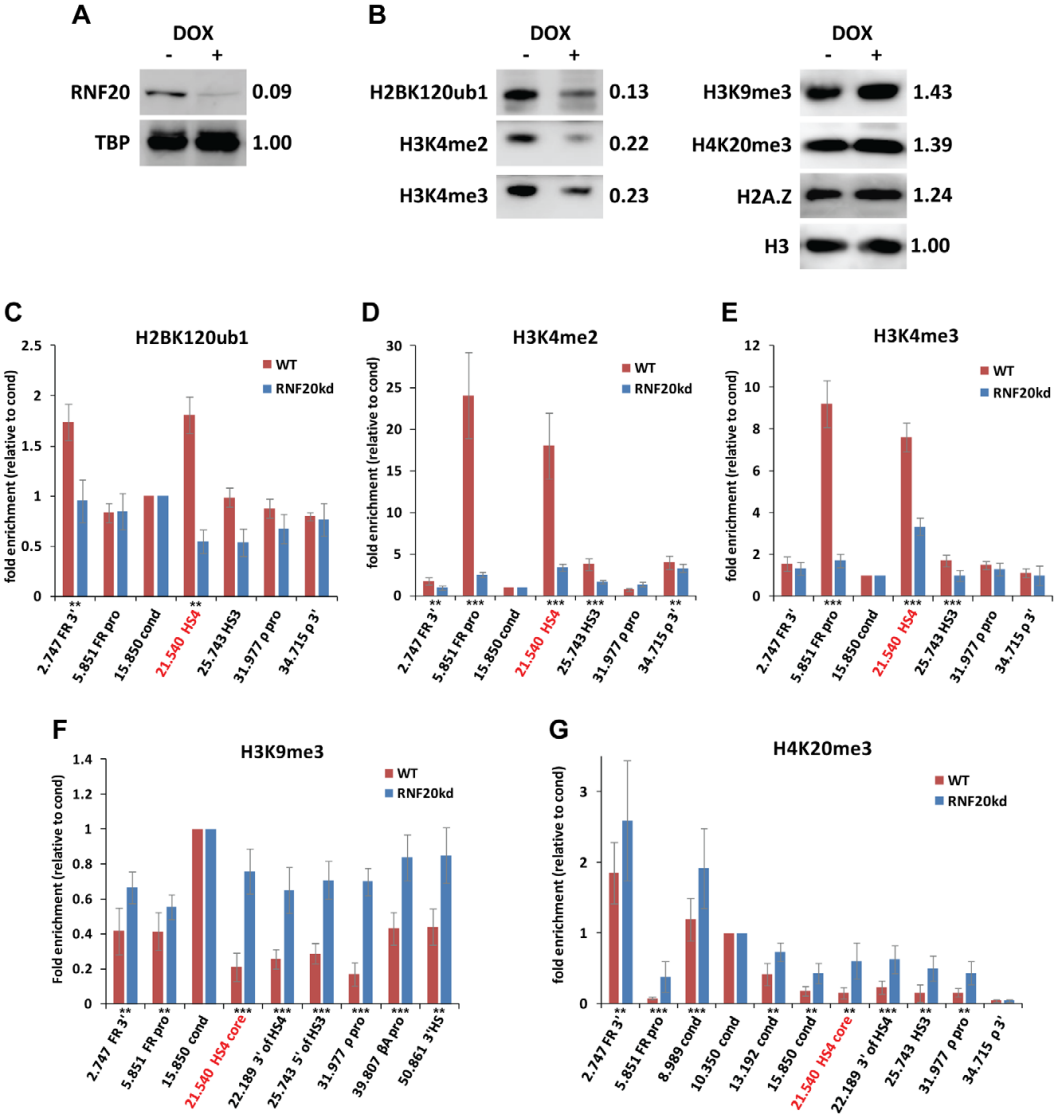
Long term loss of H2B ubiquitination results in a breach of the HS4 chromatin boundary. Analyses of early erythroid 6C2 cells following forty days of doxycycline-induced knockdown of RNF20 expression. A) Western blotting of whole cell extracts with (+) and without (−) doxycycline-induced RNF20 knockdown. The expression level of RNF20 following knockdown (relative to the loading control TBP) is shown. B) Western blotting of histone modifications present on total nucleosomes with and without RNF20 knockdown. The levels of each modification after RNF20 knockdown (relative to unmodified H3) are shown. C–G) Native ChIP analyses following forty days of RNF20 knockdown. Enrichments of C) H2BK120ub1, D) H3K4me2, E) H3K4me3, F) H3K9me3 and G) H4K20me3 at sites across the chicken *β-globin* gene neighborhood in wild type (red bars) and RNF20 knockdown (blue bars) cells. The enrichment of each sequence is normalized to the background observed at the condensed region (15.850). Significant changes in ChIP enrichments following RNF20 depletion are represented by asterisks (⋆ =  p<0.05, ⋆⋆ =  p<0.01 and ⋆⋆⋆ =  p<0.005). The location of the core HS4 insulator (21.540) is highlighted in red.

It has been postulated that the HSA/HSB regulatory region and the HS4 insulator might form chromatin boundaries that protect the *FOLR1* and *β-globin* genes from the encroachment of the potentially repressive condensed chromatin that separates these loci [15], [24]. We have therefore studied how long term depletion of H2Bub1 impacts on the containment of heterochromatin associated marks at these loci. We maintained the induction of RNF20 knockdown for forty days, which reduced proteins levels to 9% of wild type, compared to 19% seen after four days of knockdown (Figure 6A, Figure 2C). The prolonged RNF20 knockdown resulted in the depletion of H2BK120ub1 in total chromatin to 13% of wild type levels (Figure 6B). This in turn, resulted in considerable reductions in total H3K4me2 and H3K4me3, reduced by 78% and 77%, respectively (Figure 6B). Conversely, we observe 43% and 39% increases in the heterochromatin marks H3K9me3 and H4K20me3 in total chromatin (Figure 6B). This is in clear contrast to the unchanged levels of heterochromatin marks after short term knockdown (Figure 2D). Interestingly, the incorporation of the variant histone H2A.Z in total chromatin also increased by 24% after prolonged RNF20 depletion, perhaps to compensate for the gross shift from active to repressive chromatin across the genome (Figure 6B).

We performed N-ChIP analyses of histone modifications across the *FOLR1* and *β-globin* loci to determine the effects of long term RNF20 knockdown on chromatin domain integrity. Firstly, we confirmed that H2BK120ub1 was depleted from the HS4 insulator (Figure 6C). The levels of H3K4me2 and H3K4me3 at HS4 were greatly depleted (by 80% and 65%, respectively) as a result of the long term depletion of H2BK120ub1 (Figure 6D, 6E). The loss of active modifications at HS4 for a prolonged period results in extensive encroachment of the heterochromatin associated marks H3K9me3 and H4K20me3, which are normally restricted to the condensed region between the *FOLR1* and *β-globin* loci. Strikingly, H3K9me3 spreads beyond HS4 to encompass the entire 33 kb *β-globin* locus (Figure 6F). H3K9me3 spreading is likely to have occurred in the majority of cells in the population as the enrichment levels over the *β-globin* locus are comparable to those in the upstream condensed region. H3K9me3 spreading is also observed in the opposite direction, with significant increases in this mark over the *FOLR1* promoter and gene body (Figure 6F). Furthermore, H4K20me3 is also observed to spread from the upstream condensed region to cover the *FOLR1* gene in one direction and as far as the *ρ-globin* promoter in the other (Figure 6G).

### Loss of boundary function results in *FOLR1* gene silencing

**Figure 7:**
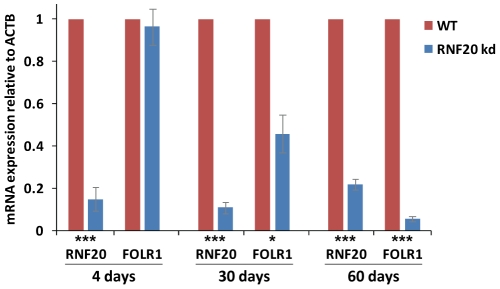
*FOLR1* expression is silenced following prolonged RNF20 depletion. Transcript levels of *RNF20* and *FOLR1* in 6C2 cells following either 4, 30 or 60 days of doxycycline-induced RNF20 knockdown. Transcript levels are normalized to β-*actin* (*ACTB*) and are shown relative to wild type 6C2 cells treated with doxycycline. Significant changes in mRNA levels in knockdown cells are represented by asterisks (⋆ =  p<0.05, ⋆⋆ =  p<0.01 and ⋆⋆⋆ =  p<0.005).

H3K9me2, H3K9me3 and H4K20me3 are widely associated with gene silencing and heterochromatin formation. The encroachment of these marks over the *FOLR1* and *β-globin* genes following RNF20 depletion may result in the silencing of their transcription. While the *β-globin* locus is becoming primed for expression at the CFU-E progenitor stage represented by 6C2 cells, the *β-globin* genes themselves are not expressed until terminal differentiation [15], [35]. 6C2 cells cannot be induced to terminally differentiate, so we are unable to study the impact of heterochromatin spreading on the activation of *β-globin* gene transcription in this system. We therefore focused our attention on the expression of the *FOLR1* gene, which is active in 6C2 cells [15]. RT-PCR analysis shows that *FOLR1* expression is not affected by four days of RNF20 knockdown (Figure 7). This is despite the depletion of H2Bub1, H3K4me2/3, H3ac, H4ac, H2A.Zac at the HSA/HSB regulatory region (Figure 3A–3D, 3F) and the encroachment of H3K9me2 across the *FOLR1* promoter and gene body (Figure 3G). Closer inspection shows that H3K4me2/3 and H4ac of the *FOLR1* promoter are unaffected following short term RNF20 depletion. *FOLR1* gene transcription is therefore not directly dependent upon RNF20 or on maximal active histone modifications at the HSA/HSB elements. However, we find that *FOLR1* gene transcription is progressively silenced with prolonged RNF20 knockdown, with 94% repression observed after sixty days of knockdown (Figure 7). The silencing of *FOLR1* coincides with both the loss of H3K4me2/3 at its promoter (Figure 6D and 6E) and the accumulation of H3K9me3 and H4K20me3 over its promoter and gene body upon heterochromatin spreading (Figure 6F and 6G).

Taken together, these findings demonstrate that the elements HSA/HSB and HS4 form the boundaries of the condensed chromatin region between the *FOLR1* and *β-globin* gene loci. They employ an H2Bub-dependent active chromatin signature that protects these genes from the encroachment of multiple heterochromatin associated marks. The spreading of H3K9me3 and H4K20me3 coincides with the silencing of the *FOLR1* gene.

## Discussion

### Similar histone signatures at the *FOLR1* and *β-globin* loci heterochromatin boundaries

**Figure 8:**
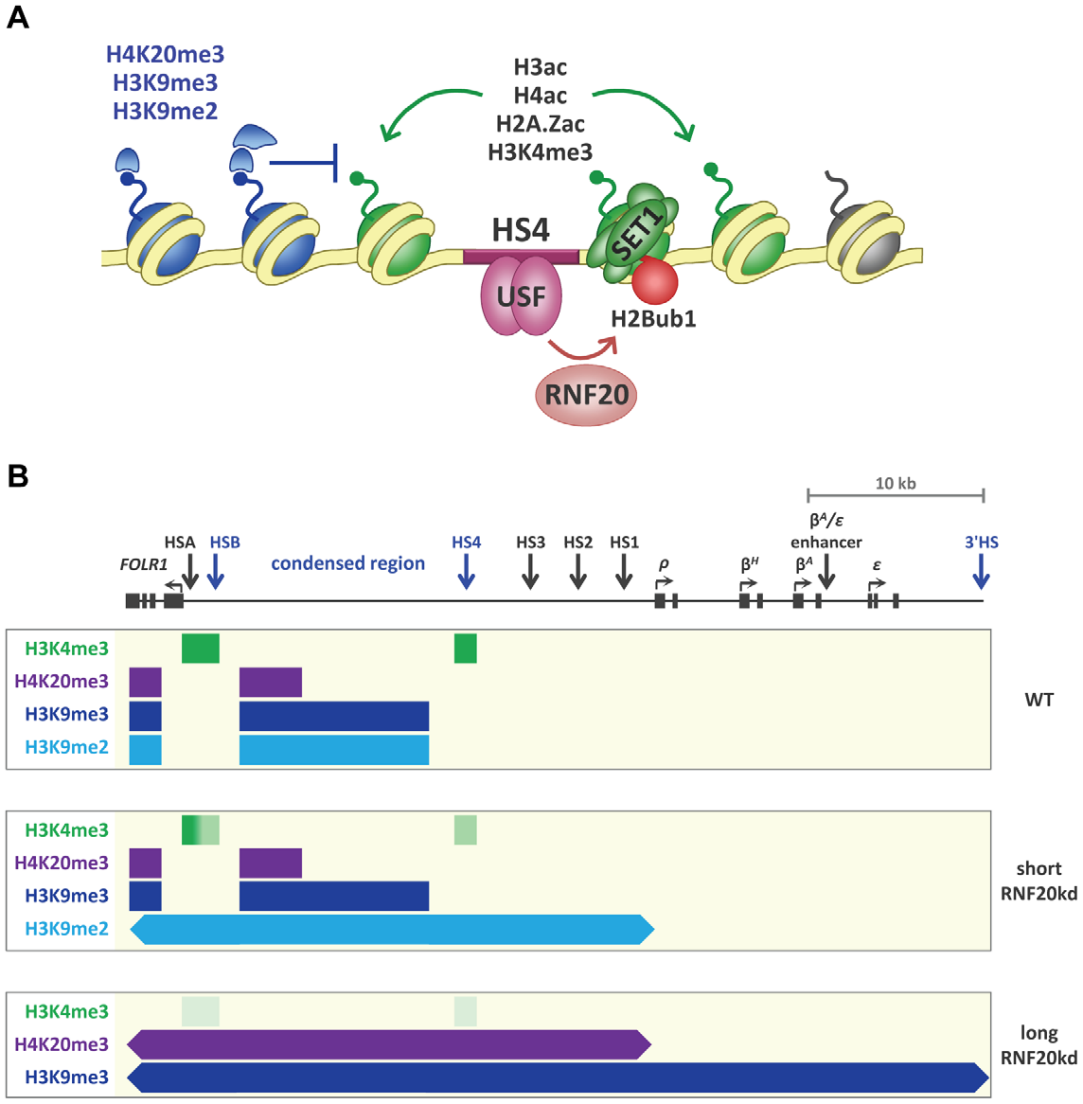
H2B ubiquitination in the establishment of a chromatin boundary. A) HS4 lies at the boundary between the *β-globin* locus and an upstream region of condensed chromatin enriched in multiple repressive histone marks (blue). H2B ubiquitination at the HS4 insulator is mediated by the E3 ligase RNF20 and is dependent upon the USF binding site. H2Bub1 is required for H3K4me3 mediated by the SET1 complex and multiple histone acetylation at two or three nucleosomes around HS4 (green). B) Scale schematic diagram summarizing the extent of chromatin domains at the *FOLR1* and *β-globin* loci mapped in wild type 6C2 cells (top panel) and cells following short-term (middle panel) and long-term (bottom panel) depletion of RNF20. Sequences enriched with H3K4me3, H4K20me3, H3K9me3 and H3K9me2 are represented by green, purple, dark blue and light blue rectangles, respectively. Faded green depicts the depletion of H3K4me3 at the boundary elements following RNF20 knockdown. Arrowheads depict the encroachment of heterochromatin marks.

The first high resolution maps of histone modifications across gene loci during vertebrate development revealed that the well characterized chromatin boundary marked by the HS4 insulator is constitutively enriched with histone modifications associated with open chromatin [13]–[14]. Here we show that the HS4 insulator is also constitutively marked by H2BK120 mono-ubiquitination. We show that RNF20-dependent H2Bub1 is required not only for H3K4me2/3 at HS4, but also for multiple acetylation of H3, H4 and H2A.Z at this element (Figure 8A). A very similar H2Bub1-dependent active histone signature is also found at the HSA/HSB elements upstream of the *FOLR1* gene. To our knowledge, this is the first example of H2Bub1 directing such an extensive cascade of *trans* histone tail modifications at specific gene regulatory elements.

HSA/HSB and HS4 mark the 5′ and 3′ flanks of the condensed chromatin region between the *FOLR1* and *β-globin* loci, which is enriched in the epigenetic hallmarks of heterochromatin ([13]–[15], this study) (Figure 8B). The loss of the active histone modification signature at these elements following the depletion of H2Bub1 in erythroid cells results in the progressive spreading of multiple repressive histone marks across the entire *FOLR1* and *β-globin* loci. These findings clearly demonstrate that the elements HSA/HSB and HS4 form the boundaries of the condensed chromatin region between the *FOLR1* and *β-globin* gene loci. The ability of the HS4 insulator to shield transgenes from chromosomal silencing in a wide variety of systems is well established [38], but this study provides firm evidence that HS4 functions as a chromatin boundary element in its endogenous context. Both the endogenous and transgenic HS4 elements require continued deposition of H2Bub1 to maintain chromatin boundary integrity and chromosomal position effect protection, respectively.

It has been unclear for some years how the *FOLR1* gene locus is defended from heterochromatin spreading. An earlier study demonstrated that a 3.7 kb region that encompasses the *FOLR1* promoter and upstream regulatory elements is capable of directing strong copy number-dependent expression of randomly integrated transgenes in chicken erythroid cells [15]. This fragment contains the major promoter-proximal element HSA and an additional DHS, which we have named HSB (Figure 1F). The elements may harbor locus control region (LCR)-like enhancer and/or chromatin boundary activities. Our observations are consistent with the latter. We find that the HSA and HSB elements are bound by the HS4 barrier proteins USF1 and VEZF1, they recruit RNF20 and the SET1 complex and establish an H2B-dependent active histone modification signature. These molecular features mirror those at the HS4 element. A key different between the HSA/HSB and HS4 boundary elements is the absence of CTCF binding at the *FOLR1* boundary. This indicates that CTCF is not required to act a barrier to the spreading of heterochromatin from the condensed region. This is consistent with our previous findings that the CTCF binding site of the HS4 insulator is dispensable for its ability to act as a barrier to chromosomal silencing in different assay contexts [18], [20].

The modification of histones at chromatin boundaries is conserved across eukaryotes. It is well established that several histone acetyltransferases (HATs) are required for heterochromatin boundary integrity in budding yeast [10], [39]–[40]. Indeed, artificial tethering of HAT chimeras is sufficient to create synthetic barriers to heterochromatin-mediated gene silencing [41]. It has also recently been found that the ILB barrier element at the *Drosophila reaper* locus also recruits histone acetylation [42]. Our observations that the depletion of multiple histone acetylation marks results in chromatin boundary failure at the chicken *FOLR1* and *β-globin* loci adds further support for a conserved role for active histone modification in chromatin boundary formation. The finding that multiple active histone modifications at the HSA/HSB and HS4 elements are directly or indirectly dependent upon prior H2B ubiquitination is particularly striking. Given the conservation of the factors that mediate H2B ubiquitination and the *trans*-histone H2Bub1-H3K4me3 pathway, we anticipate that this modification will be employed at boundaries across eukaryotes. The finding that artificial tethering of Lge1, a factor required for H2B ubiquitination and H3K4/K79 methylation, is sufficient to create a synthetic barrier to heterochromatin-mediated gene silencing in budding yeast supports this view [41], [43].

A number of budding yeast boundary elements are also associated with regions of nucleosome depletion and elevated histone turnover [40], [44]–[45]. This may be related to the incorporation of the histone variant H2A.Z, which supports heterochromatin boundary integrity [10], [46]. However, we did not observe any extensive depletion in histone density at the chicken HSA/HSB or HS4 chromatin boundaries (not shown). Furthermore, we found that the incorporation of H2A.Z at these boundaries remains intact following RNF20 knockdown and the loss of active modifications. Further studies are required to determine the role of H2A.Z at these elements, but it is clear that H2A.Z incorporation is not sufficient to prevent the spread of heterochromatin into the *FOLR1* and *β-globin* loci.

### Histone crosstalk and chromatin boundary formation

We have shown that the *trans* histone modification pathway from H2Bub1 to H3K4me3 reported in yeast and man is also conserved in chicken. How the mono-ubiquitination of H2B facilitates H3K4me3 has been subject to intense study over the last few years. Three models have arisen to explain this pathway. The ‘wedge’ model postulated that the bulky ubiquitin moiety would increase the access of H3K4 methyltransferases by non-specifically disrupting chromatin fiber packing in some way [47]–[48]. This simple mechanism appears improbable as substitution of ubiquitin with the bulkier SUMO moiety at the equivalent residue of H2B does not recapitulate H2Bub1-directed *trans* tail crosstalk in *S. cerevisiae* [49]. In contrast, a ‘stability’ model was recently put forward in response to findings in *S. cerevisiae* that H2Bub1 promotes nucleosome reassembly following RNA polymerase II transcription and enhances global nucleosome stability [49]–[51]. It is proposed that H2Bub1 may restrict the eviction of the H2A/H2B dimer from nucleosomes, thereby increasing the nucleosomal residence of the SET1/COMPASS methyltransferase complex which interacts with basic and acidic patches on H2A and H2B, respectively [52]–[53]. In this study, we found that the interaction of RBBP5 (SWD1), a core component of the SET1 complex remains bound at the HS4 insulator following the depletion of H2Bub1. The loss of H3K4me2/3 cannot be explained by the decreased residence of SET1 complexes.

Recent studies provide compelling evidence that H2Bub1 acts as ‘bridge’ to facilitate H3K4me3. The core SET1 complex can interact with chromatin and mediate H3K4 mono-methylation in the absence of H2B ubiquitination [54]. However, it has been found that the accessory COMPASS subunit Cps35/Swd2 in yeast (WDR82 in humans) interacts with H2Bub1 and activates the processive H3K4 methyltransferase activity of the SET1 complex [55]–[56]. While the composition of SET1 complexes in chickens remains to be determined, our data are consistent with a mechanism of H2Bub1-directed activation of pre-loaded SET1 complexes to facilitate processive H3K4 methylation. The loss of H3K4me2/3 upon the depletion of H2Bub1 is likely to be the primary reason for the subsequent losses of multiple histone acetylation at the HS4 insulator. Methylated H3K4 is a pivotal recognition site required by multiple histone acetyltransferase complexes [57]–[60]. H3K4me3 also facilitates the recruitment of the NURF chromatin remodelling complex via its BPTF subunit [22], [61].

### *Trans* factor-directed H2B ubiquitination at chromatin boundaries

The mono-ubiquitination of H2B is broadly recognized as a mark of transcriptional activity [30]. H2Bub1 is enriched in the bodies of expressed genes throughout yeast and mammalian genomes [62]–[63], and the bulk of H2Bub1 requires many factors involved in the early steps of transcription elongation [30]. While the HS4 insulator has the epigenetic chromatin signature of a housekeeping promoter, it lacks either promoter or enhancer activity [64]. In addition, HS4 is not bound by RNA polymerase II (Figure S4) and is not a source of transcripts [22], [65]. It therefore appears most likely that HS4 recruits H2Bub1 through a process that is not linked to transcription. We found that the recruitment of H2Bub1 to HS4 is dependent upon the USF1/USF2 binding site. While we have been unable to detect RNF20 in stable complexes with USF1 or USF2 (data not shown) [22], this is reminiscent of activator-dependent recruitment of Bre1/RNF20 to yeast and human promoters [66].

In addition to the recruitment of the E3 ubiquitin ligase RNF20, the HSA/HSB and HS4 boundary elements also require sufficient activity levels of the E2 conjugase RAD6 to enable sufficient levels of H2Bub1 for chromatin boundary stability. There are two broad mechanisms that could result in the persistent H2B ubiquitination of the HS4 insulator. Firstly, the HSA/HSB and HS4 elements might not be subject to the rapid turnover of H2Bub1 associated with promoter clearance and transcription elongation [66]–[67]. Such a scenario would negate the need for the co-transcriptional stimulation of RAD6 conjugase activity [68], as low efficiency H2B ubiquitination may be sufficient for high steady state levels of H2Bub1 at HS4. Alternatively, HS4 may recruit factors that mediate RAD6 phosphorylation in the absence of RNA polymerase to stimulate efficient H2B ubiquitination of this element.

### Heterochromatin spreading beyond defective boundaries

Depletion of H2Bub1 disrupts the assembly of the active histone modification signatures at the HSA/HSB and HS4 boundary elements. This results in progressive spreading of heterochromatin-associated histone marks into the *FOLR1* and *β-globin* loci either side of the condensed region. We find that the heterochromatin-associated marks H3K9me2, H3K9me3 and H4K20me3 are propagated in a continuous manner from the upstream condensed region into the *FOLR1* and *β-globin* loci. The heterochromatin domain expands from a ∼10 kb domain of the condensed region to cover the entire ∼50 kb *FOLR1* and *β-globin* region given time (Figure 8B).

Intriguingly, each of the three repressive marks at the *β-globin* locus spreads in a different temporal manner, suggesting that different enzyme complexes are involved in propagating these marks. The first repressive mark to spread is H3K9me2, which propagates over the entire *FOLR1* locus and extends 14 kb into the *β-globin* locus after only four days of H2Bub1 depletion (Figure 8B). Conversely, H3K9me3 and H4K20me3 do not extend beyond the upstream condensed region at this early stage, but H3K9me3 appears to consolidate at the borders of the condensed region. However, both H3K9me3 and H4K20me3 spread into the *FOLR1* and *β-globin* loci upon longer periods of H2Bub1 depletion. H3K9me3 uniformly spreads to encompass the entire ∼50 kb *FOLR1* and *β-globin* region, while H4K20me3 spreads into a ∼ 30 kb region covering the entire *FOLR1* locus to the *rho* gene promoter (Figure 8B).

Several mechanisms have been proposed to explain the spreading of repressive chromatin [69]. Given that the *de novo* repressive marks in the *FOLR1* and *β-globin* loci manifest as continuous domains with consistent modification levels throughout, we speculate that the marks are propagated via linear *cis*-spreading mechanisms. The simplest way to rationalize all our findings is that the spreading occurs using a classical stepwise assembly mechanism, where sequential iterations of repressor protein binding and methyltransferase recruitment propagate the repressive methyl mark onto neighboring nucleosomes. The ability of HP1 adaptor proteins to recognize H3K9me3, interact with H3K9 and H4K20 methyltransferases and spread from sites of recruitment is a potential example of the self-reinforcing repressor interactions that may occur at the *FOLR1* and *β-globin* loci [70]–[72]. Stepwise assembly mechanisms are consistent with the observed sequential pathway of repressive chromatin modification. It is possible that the propagation of H4K20me3 might require prior H3K9me3, which requires prior H3K9me2 at this locus. While further investigations will be required to define the exact pathway of repressive mark assembly, it is clear that HS4 acts a chain terminator to heterochromatin spreading by using a panel of active histone modifications, which collaborate to block and inhibit repressive histone methylation.

There may be a role for RNA-directed heterochromatin assembly at the *FOLR1* and *β-globin* loci. It was recently shown that the maintenance of heterochromatin region's condensed conformation requires RNAi factors [65]. This suggests conservation with mechanisms employed in fission yeast where RNAi factors work together with heterochromatin proteins including the HP1 homolog Swi6 to mediate heterochromatin establishment [8]. However, RNAi factors are dispensable for the maintenance of heterochromatin [73]. It remains to be investigated whether RNA and RNAi factors play a role in heterochromatin spreading in higher eukaryotes.

It is also conceivable that the continuous spreading of heterochromatin is dictated by the three dimensional organization of the *FOLR1* and *β-globin* loci. If these gene loci are insulated from the upstream condensed region by positioning into different nuclear compartments, disruption of the active signature at the HSA/HSB and HS4 boundaries may result in the transfer of most or all the *FOLR1* and *β-globin* loci into a repressive compartment. Such a scenario appears complex, however, as it would require the sequential transfer of these loci into different compartments rich in H3K9me2, H3K9me3 and H4K20me3 methyltransferases.

There is a paucity of information about the elements that form chromatin boundaries in vertebrates. Our finding that the HSA/HSB and HS4 boundary elements employ H2B ubiquitination to direct a cascade of active histone modifications suggests that genomic profiling of chromatin signatures will be a useful approach to identifying boundary elements. We note that there was a gross increase in total heterochromatin marks results when H2Bub1 is depleted for long periods. This observation suggests that many loci employ H2Bub1-dependent boundaries to heterochromatin spreading. It will be interesting to see whether other chromatin boundaries in vertebrate genomes also require such a large complement of active marks or employ a more restricted palette to deal with locus-specific threats.

## Materials and Methods

### Antibodies

Antibodies against H3K4me2 (07-030), H3K4me3 (05-745R), H3K9me3 (07-523), H3K27me3 (07-449), H3 (07-690), H3K9acK14ac (06-599), H4K5acK8acK12acK16ac (06-598), H2AK119ub (05-678), H2A.Z (07-594) and CTCF (06-917) were obtained from Millipore. Antibodies against H3K9me2 (ab1220), H3K79me2 (ab3594), H3K79me3 (ab2621), H2A.ZK4acK7acK11ac (ab18262), PAF1 (ab20662), RPB1 (ab5408) and TBP (ab51841) were obtained from Abcam. Antibodies against H4K20me3 were a kind gift from Judd Rice [74]. Antibodies against ubiquitin (sc-8017), (BML-PW8805) and USF1 (H00007391-A01) were obtained from Santa Cruz, Enzo Life Sciences and Abnova, respectively. Antibodies against RNF20 (A300-715A) and RBBP5 (A300-109A) were obtained from Bethyl Laboratories. Anti-H2BK120ub1 antibodies were initially a kind gift from Moshe Oren [62] then purchased from Médimabs (MM-0029) or Millipore (17-650). Anti-VEZF1 antibodies were raised as described [21]. PE conjugated anti-CD25 (IL-2R) was obtained from Dako.

### Crosslinking chromatin immunoprecipitation

Chicken 6C2 erythroleukaemia cells were grown in αMEM supplemented with 10% FCS, 2% chicken serum, 1 mM HEPES, 25 µM β-mercaptoethanol and 1% Penicillin/Streptomycin solution. Crosslinking chromatin immunoprecipitation was performed as described previously [Litt *et al*, 2001]. Briefly, 6C2 cells (2×10^7^ cells/ml) were crosslinked in fresh growth medium with 1% formaldehyde at room temperature for 20 minutes (RNF20, PAF1 and RBBP5), 10 minutes (CTCF, USF1 and VEZF1) or 2 minutes (H2Bub). Reactions were quenched by adding glycine to a final concentration 0.125 M. The crosslinked cells were washed by PBS twice and then lysed (0.25% Triton X-100, 10 mM EDTA, 0.5 mM EGTA and 10 mM Tris pH 8). Cell nuclei collected by centrifugation were washed (0.2 M NaCl, 1 mM EDTA, 0.5 mM EGTA and 10 mM Tris pH 8) followed by chromatin solubilization (0.5% SDS, 10 mM EDTA and 50 mM Tris pH 8). Chromatin was fragmented by sonication (Misonix) for a total time of 10 minutes in regular 10 second pulses. Insoluble material was removed by centrifugation at 15,000 g for 10 minutes at 4°C. Sizes of chromatin fragments were ∼500 bp on average.

Soluble chromatin was diluted by X-ChIP buffer (1.1% Triton X-100, 1.2 mM EDTA, 167 mM NaCl, 0.01% SDS and 16.7 mM Tris pH8) to obtain chromatin from 1×10^7^ cells per ml. 1 ml of chromatin was pre-cleared with 5 µg of non-immune IgG and 100 µl (50% slurry in X-ChIP) of protein A/G agarose at 4°C for 3 hours. 10 µg of specific antibody was incubated with pre-cleared chromatin at 4°C with agitation overnight. Binding of protein A/G agarose was carried out at 4°C for 2 hours. The agarose was washed extensively with buffer 1 (1% Triton X-100, 0.1% SDS, 2 mM EDTA, 150 mM NaCl and 20 mM Tris pH 8), buffer 2 (1% Triton X-100, 0.1% SDS, 2 mM EDTA, 500 mM NaCl and 20 mM Tris pH 8), buffer 3 (0.25 M LiCl, 1% NP-40, 0.5% sodium deoxycholate, 1 mM EDTA, 10 mM Tris pH 8) and twice with TE buffer (10 mM Tris pH 8, 1 mM EDTA). The bound chromatin was eluted into elution buffer (1% SDS, 0.1 M NaHCO_3_), crosslinks reversed and protein digested. DNA was extracted by phenol/chloroform and ethanol precipitated in the presence of 10 µg of glycogen for quantitative PCR (qPCR) analysis.

### Low-salt native chromatin immunoprecipitation

Circulating red blood cells and whole brain tissue (without major vasculature) were collected from 10 day fertilized chick embryos kindly provided by Aviagen, Ltd. Native nucleosomes were prepared in low salt conditions to ensure retention of all nucleosomes, as described [34]. In brief, cells were collected in the presence of inhibitors (25 µg/ml AEBSF, 0.5 µg/ml Leupeptin and 0.7 µg/ml Pepstatin, 10 mM N-ethylmaleimide and 10 mM sodium butyrate) and nuclei were isolated by lysis buffer (10 mM NaCl, 3 mM MgCl_2_, 0.4% NP-40 and 10 mM Tris pH 7.5) for MNase (Sigma) digestion in the presence of 1 mM CaCl_2_. The MNase concentration (X) required to yield mostly di- and tri-nucleosomes was firstly determined. For ChIP experiments, three equal aliquots of nuclei were incubated with ½X, 1X and 2X MNase at 37°C for 17 minutes to obtain representative di- and tri-nucleosomes [14]. Digestion was stopped with 10 mM EDTA. Soluble chromatin was collected by centrifugation at 2,500 g for 5 minutes. The three supernatants were combined (S1). The remaining pellets were combined and resuspended in lysis buffer supplemented with 10 mM EDTA and left on ice for 15 minutes. Chromatin was released by passing through 20 then 25 gauge needles, and collected by centrifugation at 10,000 g for 10 minutes. The supernatant (S2) was combined with S1 for sucrose gradient fractionation. ∼1.5 mg of S1–S2 chromatin was fractionated on 13.5 ml 5∼25% linear sucrose gradients (Biocomp gradient master) in a SW40Ti rotor at 31,000 rpm for 14 hours at 4°C. 1 ml fractions were collected and 10 µl aliquots were extracted for checking DNA fragment sizes. Fractions containing di- and tri-nucleosomes were pooled and fixed with 0.1% formaldehyde at room temperature for 10 minutes. The crosslinking reaction was stopped with 0.125 M glycine. Nucleosomes were exchanged into N-ChIP buffer (50 mM NaCl, 5 mM EDTA, 10 mM Tris pH 7.5) buffer using P-6DG Bio-Gel (BioRad).

50 µg of nucleosomes were pre-cleared with 5 µg of non-immune IgG and 100 µl (50% slurry in N-ChIP buffer) of protein A/G agarose at 4°C for 3 hours. 10 µg of specific antibody was incubated with pre-cleared chromatin at 4°C with agitation overnight. Binding of protein A/G agarose was carried out at 4°C for 2 hours. Immunoprecipitated chromatin was collected and washed 5 times with 1 ml N-ChIP wash buffer (150 mM NaCl, 0.2 mM EDTA, 0.1% Tween-20 and 20 mM Tris pH 7.4). Chromatin was eluted with N-ChIP buffer supplemented with 1% SDS followed by 0.5% SDS. Eluates were digested with Proteinase K at 45°C for 2 hours and DNA extracted by phenol/chloroform and precipitated for qPCR analysis.

### Quantitative PCR analysis

Relative DNA enrichments were quantified in triplicate by TaqMan real-time PCR on a Roche 480 Lightcycler. The primers used in this study were described previously [14], [21]. The comparative Ct method (with correction for primer efficiencies) was used to calculate fold enrichments and their standard deviations, as described previously [24]. Two sample equal variance Student's t-tests using a two-tailed distribution were applied to ChIP enrichment values to assess the significance of enrichments over controls, or changes following RNF20 knockdown. The calculated p-value ranges for enriched sites are indicated in the figure legends.

### RNA interference

The pSLIK micro RNA-based lentiviral expression system was used to mediate long term conditional knockdown of RNF20 in chicken cells [75]. Gene-specific shRNAs are embedded into the primary transcript of human miR30, which is located in the 3′UTR of a doxycycline-regulated GFP transgene. The psm2 shRNA design tool was used to identify 20 potential shRNA targets (http://hannonlab.cshl.edu). These were scored and four targets were cloned into pEN_hUmiRc2, packaged into lentivirus particles and tested for performance as previously described [21]. GgRNF20-2628, which targets CAGAGTAACTAGAGAGAAA, was the most potent miR-shRNA and was used throughout this study. Wild type or 8103 (containing a single copy HS4 flanked *IL-2R* reporter transgene, [24]) 6C2 cells were transduced with GgRNF20-2628 lentiviral particles and cloned following flow sorting or serial dilution. GFP-miRNA expression was induced with 2 µg/ml doxycycline. GFP expression was monitored by FACS analysis to confirm expression of the miRNA cassette and RNF20 protein levels were monitored by Western blotting during prolonged knockdown time courses.

### Western blotting

Nuclear extracts were prepared from cells harvested and washed with PBS and then lysed with hypotonic buffer (0.2% NP-40, 0.1 mM EDTA and 20 mM HEPES pH 8). Cell nuclei were collected by centrifugation at 2,500 g for 5 minutes. Nuclear proteins were extracted by incubation in high salt buffer (0.2% NP-40, 0.4 M NaCl, 13.3% glycerol and 20 mM HEPES pH 8) at 4°C for 30 minutes. Insoluble debris was removed by centrifugation at 16,000 g for 10 minutes at 4°C. Soluble nuclear extract was quantified by Bradford assay (Bio-Rad). 25 µg of nuclear extract or 7 µg of native S1–S2 nucleosome preparations were used for separation by SDS-PAGE. Proteins were then transferred to a PVDF membrane and imaged with HRP-conjugated secondary antibodies on a LAS-3000 imager (Fujifilm). Band intensities corrected for background were quantified using AIDA software.

### FACS analysis

10^6^ cells were harvested by centrifugation at 1,000 g for 5 minutes. Cells were washed twice in 1 ml of Hank's buffered saline solution (Sigma) supplemented with 0.1% BSA and 0.1% NaN_3_ (HBSS+). Cells were resuspended in 100 µl of HBSS+ and incubated with 10 µl of anti-CD25-PE (Dako) antibody in the dark at 4°C for 30 minutes. Excess antibody was removed by washing twice with 1 ml of HBSS+. After the last wash, cells were resuspended in 500 µl of HBSS+ for FACS analysis. Flow cytometry was performed on a FACSCalibur flow cytometer (BD Biosciences) using CELLQuest software. RNF20 knockdown cells express GFP upon induction with doxycycline. Color compensation was used to correct for GFP fluorescence in the FL2 (585 nm) filter; it was set as FL1 – 1% FL2 and FL2–20% FL1. Data was acquired for 10,000 viable or GFP-expressing cells (FL1  =  530 nm). Histograms were generated using FlowJo software (Tree Star, Inc). Mean IL-2R fluorescence intensities of RNF20 knockdown cells were determined and normalized to those of parental reporter transgene cells with wild type RNF20 levels.

### RT-PCR

Total RNA was isolated from 6C2 cells using TRI reagent (Sigma) and cDNA prepared using SuperScript III (Invitrogen) and random hexamers. The following primers were used SYBR green real time PCR assays:

5′ TGCTGCGCTCGTTGTTGA

5′ CATCGTCCCCGGCGA

5′ ATGCGTCATCTCATCAGCAG

5′ TTGGGAAGAAGGGTCATCAG

5′ GATTCTGCATGTGCCACTGT

5′ AAGACCTGGGTGAAGGGTCT

## Supporting Information

Figure S1 Knockdown of chicken RNF20. A) Pairwise alignment (http://multalin.toulouse.inra.fr/multalin) of human (NP_062538.5) and chicken (NP_001026605.1) RNF20/BRE1A. The conserved RING domain is boxed. The RNF20 antibodies used in this study were raised against a conserved epitope between residues 125 and 175. B) RT-PCR analysis of RNF20 knockdown following doxycycline induction of shRNA expression. C) Western blotting of 6C2 whole cell extracts before and after doxycycline-induced RNF20 RNAi. Blots probed with anti-RNF20 (expected size of 120 kDa) or anti-TBP (expected size of 38 kDa) are shown with the positions of molecular weight markers (kDa).
(TIF)

Figure S2 The ubiquitin ligase RNF20 mediates H2B ubiquitination at the HS4 insulator. Native ChIP of H2BK120 monoubiquitination at sites across the chicken *β-globin* gene neighborhood in wild type (red bars) and RNF20 knockdown (blue bars) 6C2 cells. The enrichment of each sequence is normalized to the background observed downstream of the inactive *ρ-globin* gene. Significant changes in ChIP enrichments following RNF20 depletion are represented by asterisks (⋆ =  p<0.01, ⋆⋆ =  p<0.001 and ⋆⋆⋆ =  p<0.0001).
(TIF)

Figure S3 Loss of H2B ubiquitination does not initially affect H4K20me3 containment. Native ChIP analyses of early erythroid 6C2 cells following four days of doxycycline-induced knockdown of RNF20 expression. Enrichments of H4K20me3 at sites across the chicken *β-globin* gene neighborhood in wild type (red bars) and RNF20 knockdown (blue bars) cells. The enrichment of each sequence is normalized to the background observed at the condensed region (15.850). The location of the core HS4 insulator (21.540) is highlighted in red. Significant changes in ChIP enrichments following RNF20 depletion are represented by asterisks (⋆ =  p<0.05, ⋆⋆ =  p<0.01 and ⋆⋆⋆ =  p<0.005). The location of the core HS4 insulator (site 21.540) is highlighted in red.
(TIF)

Figure S4 RNA polymerase II binding at the transcriptionally active *FOLR1* gene in 6C2 cells Crosslinking ChIP analysis of RNA polymerase II (RPB1 CTD, all forms) occupancy at the chicken *FOLR1*/*β-globin* loci in 6C2 cells. Significant ChIP enrichments are represented by asterisks (⋆ =  p<0.01, ⋆⋆ =  p<0.001 and ⋆⋆⋆ =  p<0.0001).
(TIF)
