# Supplementary figures and images for: Histone Crosstalk Directed by H2B Ubiquitination Is Required for Chromatin Boundary Integrity
Source: PLoS Genet. 2011 Jul 21;7(7):e1002175. doi: 10.1371/journal.pgen.1002175 (PMC3140996; doi:10.1371/journal.pgen.1002175)

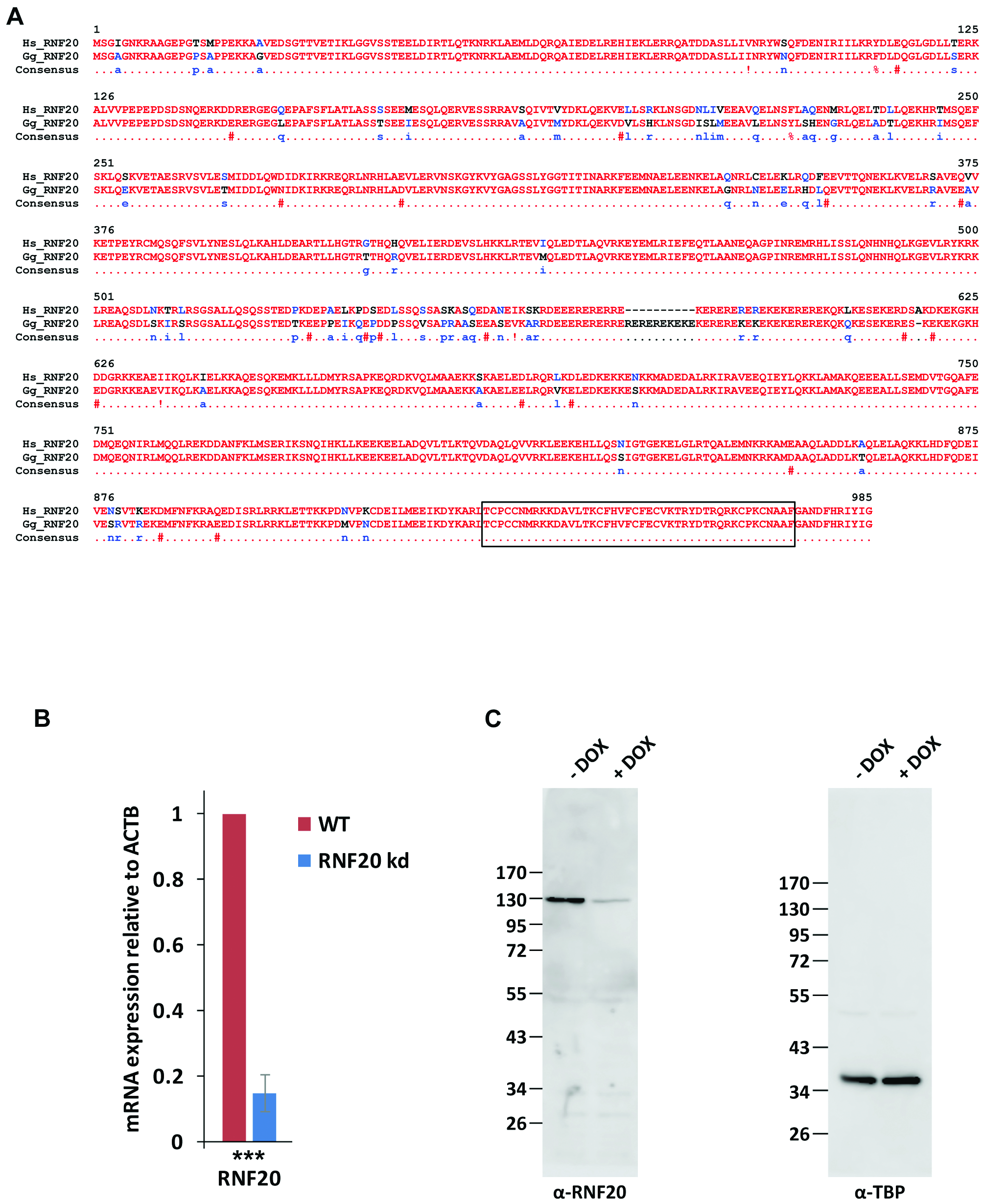

Supplement: Figure S1 — Knockdown of chicken RNF20. A) Pairwise alignment (http://multalin.toulouse.inra.fr/multalin) of human (NP_062538.5) and chicken (NP_001026605.1) RNF20/BRE1A. The conserved RING domain is boxed. The RNF20 antibodies used in this study were raised against a conserved epitope between residues 125 and 175. B) RT-PCR analysis of RNF20 knockdown following doxycycline induction of shRNA expression. C) Western blotting of 6C2 whole cell extracts before and after doxycycline-induced RNF20 RNAi. Blots probed with anti-RNF20 (expected size of 120 kDa) or anti-TBP (expected size of 38 kDa) are shown with the positions of molecular weight markers (kDa). (TIF) [file pgen.1002175.s001.tif]

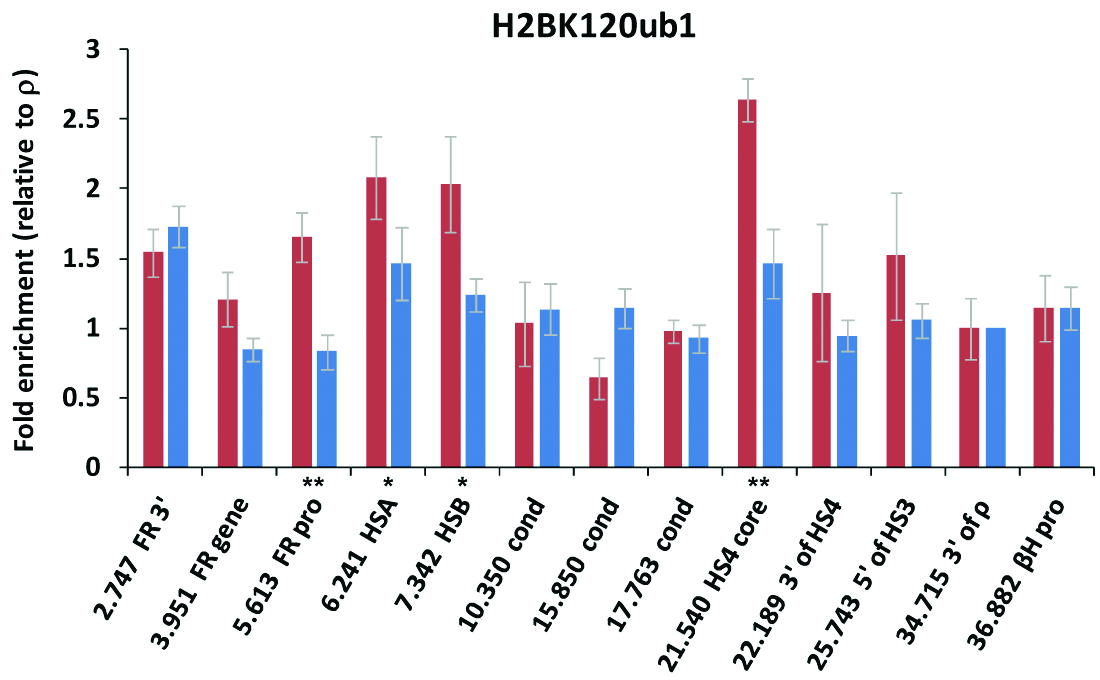

Supplement: Figure S2 — The ubiquitin ligase RNF20 mediates H2B ubiquitination at the HS4 insulator. Native ChIP of H2BK120 monoubiquitination at sites across the chicken β-globin gene neighborhood in wild type (red bars) and RNF20 knockdown (blue bars) 6C2 cells. The enrichment of each sequence is normalized to the background observed downstream of the inactive ρ-globin gene. Significant changes in ChIP enrichments following RNF20 depletion are represented by asterisks (⋆ = p<0.01, ⋆⋆ = p<0.001 and ⋆⋆⋆ = p<0.0001). (TIF) [file pgen.1002175.s002.tif]

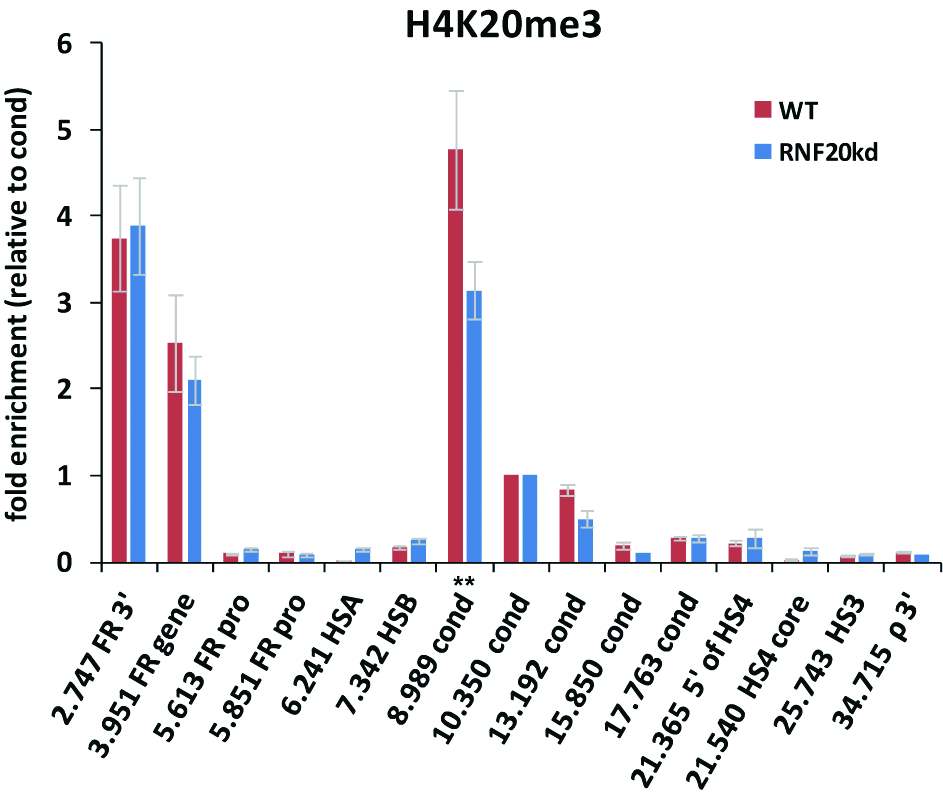

Supplement: Figure S3 — Loss of H2B ubiquitination does not initially affect H4K20me3 containment. Native ChIP analyses of early erythroid 6C2 cells following four days of doxycycline-induced knockdown of RNF20 expression. Enrichments of H4K20me3 at sites across the chicken β-globin gene neighborhood in wild type (red bars) and RNF20 knockdown (blue bars) cells. The enrichment of each sequence is normalized to the background observed at the condensed region (15.850). The location of the core HS4 insulator (21.540) is highlighted in red. Significant changes in ChIP enrichments following RNF20 depletion are represented by asterisks (⋆ = p<0.05, ⋆⋆ = p<0.01 and ⋆⋆⋆ = p<0.005). The location of the core HS4 insulator (site 21.540) is highlighted in red. (TIF) [file pgen.1002175.s003.tif]

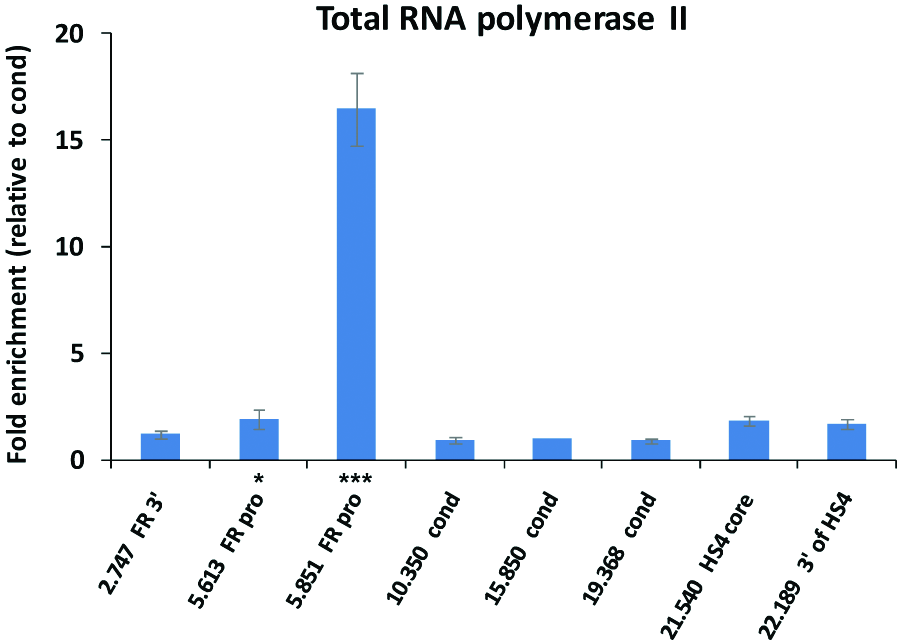

Supplement: Figure S4 — RNA polymerase II binding at the transcriptionally active FOLR1 gene in 6C2 cells Crosslinking ChIP analysis of RNA polymerase II (RPB1 CTD, all forms) occupancy at the chicken FOLR1/β-globin loci in 6C2 cells. Significant ChIP enrichments are represented by asterisks (⋆ = p<0.01, ⋆⋆ = p<0.001 and ⋆⋆⋆ = p<0.0001). (TIF) [file pgen.1002175.s004.tif]
